# Dosimetric factors and Lyman normal-tissue complication modelling analysis for predicting radiation-induced lung injury in postoperative breast cancer radiotherapy: a prospective study

**DOI:** 10.18632/oncotarget.12979

**Published:** 2016-10-28

**Authors:** Zhi-Rui Zhou, Qing Han, Shi-Xiong Liang, Xiao-Dong He, Nu-Yun Cao, Ying-Jie Zi

**Affiliations:** ^1^ Department of Radiation Oncology, Fudan University, Shanghai Cancer Center, Shanghai, P.R.China; ^2^ Department of Oncology, Shanghai Medical College, Fudan University, Shanghai, P.R.China; ^3^ Department of Radiation Oncology, Cancer Hospital of Guangxi Medical University, Nanning, P.R.China; ^4^ Department of Radiation Oncology, Shanghai Pulmonary Hospital, Tongji University School of Medicine, Shanghai, P.R.China; ^5^ College of Mathematics and Information Science, Guangxi University, Nanning, P.R.China

**Keywords:** intensity modulated radiotherapy, normal tissue complication probability, radiation-induced lung injury, breast cancer

## Abstract

To investigate the relationship between dosimetric factors, including Lyman normal-tissue complication (NTCP) parameters and radiation-induced lung injury (RILI), in postoperative breast cancer patients treated by intensity modulated radiotherapy (IMRT). 109 breast cancer patients who received IMRT between January 2012 and December 2013 were prospectively enrolled. A maximum likelihood analysis yielded the best estimates for Lyman NTCP parameters. Ten patients were diagnosed with RILI (primarily Grade 1 or Grade 2 RILI); the rate of RILI was 9.17% (10/109). Multivariate analysis demonstrated that ipsilateral lung V_20_ was an independent predictor (*P*=0.001) of RILI. Setting V_20_=29.03% as the cut-off value, the prediction of RILI achieved high accuracy (94.5%), with a sensitivity of 80% and specificity of 96%. The NTCP model parameters for 109 patients were m=0.437, *n*=0.912, and TD_50_(1)=17.211 Gy. The sensitivity of the modified Lyman NTCP model to predict the RILI was 90% (9/10), the specificity was 69.7% (69/99), and the accuracy was 71.6% (78/109). The RILI rate of the NTCP<9.62% in breast cancer patients was 1.43% (1/70), but the RILI rate of the NTCP>9.62% in patients with breast cancer was 23.08% (9/39), (*P*=0.001). In conclusion, V_20_ is an independent predictive factor for RILI in patients with breast cancer treated by IMRT; V_20_=29.03% could be a useful dosimetric parameter to predict the risk of RILI. The Lyman NTCP model parameters of the new value (m=0.437, *n*=0.912, TD50 (1) =17.211 Gy) can be used as an effective biological index to evaluate the risk of RILI.

## INTRODUCTION

Breast cancer is the most common cancer of women worldwide [1–3]. As in most other countries, the health burden of cancer is increasing in China, with more than 1.6 million people being diagnosed and 1.2 million people dying of the disease each year [2, 4, 5]. Fan Lei stated that the annual number of new cases of breast cancer and death in China accounted for 12.2% and 9.6% of cases worldwide, respectively [4]. Postoperative adjuvant radiotherapy can significantly reduce the recurrence and mortality of breast cancer and improve the quality of life for breast cancer patients [6–8].

In recent years, intensity-modulated radiation therapy (IMRT) has achieved a good curative effect in the treatment of breast cancer. In the process of breast cancer radiotherapy, lung tissue is affected by the dose of irradiation, resulting in radiation-induced lung injury (RILI) of different degrees. The main manifestation of RILI is acute radiation pneumonitis (RP) or chronic pulmonary interstitial fibrosis (PF) [9]. RILI is one of the common complications of radiotherapy after breast cancer surgery; it negatively affects patients’ quality of life and may even lead to death in cases of severe RILI [9–11]. Therefore, it is particularly important to predict the occurrence and the degree of RILI based on the successful completion of radiotherapy plans and to prevent further pulmonary radiation injuries in early stages.

Some studies have suggested that factors including age, gender, pulmonary function, lung irradiation volume and the use of chemotherapy were associated with RILI [12]. The normal tissue complication probability (NTCP) is considered to be a good predictor of RILI factors; many scholars have studied the NTCP model of pulmonary complications after radiotherapy [10, 13]. In our study, a comprehensive analysis of breast cancer patients was performed to assess factors predictive of RILI; the Lyman NTCP model parameters were explored to determine their predictive value.

## RESULTS

### Baseline characteristics and clinical outcome

From January 2012 to December 2013, a total of 109 patients met the inclusion criteria; the median follow-up time was 13 months (6 to 26 months). Patients were diagnosed with breast cancer at ages ranging from 29 to 73 years old; the median age at diagnosis was 45 years. The patients were divided into two groups based on whether RILI developed. The absence of RILI group included 99 cases, while the RILI group comprised 10 cases; the incidence was 9.17%. There were 4 cases of acute grade 1 RILI and 4 cases of acute grade 2 RILI, which occurred within the first 6 months after radiotherapy; the symptoms resolved after treatment with antibiotics and high-dose hormones. No patients experienced grade 3, 4 or 5 acute RILI. Chronic RILI was reported in 2 cases: one in grade 2 and one in grade 3. Chronic RILI occurred more than 6 months after radiotherapy. The incidence of RILI was independent of the age of patients, operation method, and clinical stage (*P* > 0.05), while the number of chemotherapy cycles was significant (χ^2^ = 5.825, *P* < 0.05). More details are shown in Table 1.

**Table 1:** The univariate analysis of radiation-induced lung injury (RILI) and clinical factors in 109 breast cancer patients

Clinicopathology factors		Lung injury group (*n*=10)	No injury group (*n*= 99)	*N*	*P value*
Age(years)	≤60	9	92	101	0.735
	>60	1	7	8	
The number of cycles of chemotherapy	≤6	2	64	66	0.016
	>6	8	35	43	
Operation method	Breast conserving surgery	5	56	61	0.949
	Modified radical mastectomy	5	43	48	
Clinical staging*	Stage I	2	29	31	0.172
	Stage II	2	41	43	
	Stage III	6	29	35	

^*^According to the seventh edition of AJCC staging criteria

### Physical parameters analysis

The average volume of ipsilateral lung (t = -4.011, *P* < 0.05), ipsilateral lung V_5_ (t = -2.771, *P* < 0.05), V_10_ (t = -3.683, *P* < 0.05), V_15_ (t = -4.113, *P* < 0.05), V_20_ (t = -4.541, *P* < 0.05), V_25_ (t = -2.747, *P* < 0.05) and the occurrence of RILI were analysed; the results are shown in Table 2.

**Table 2:** The univariate analysis of radiation-induced lung injury (RILI) and physical parameters (X±SD) in 109 breast cancer patients

Dosmetry factors	Lung injury group (*n*=99)	No injury group (*n*=10)	*P value*
V_5_ (%)	67.0±15.1	80.6±10.4	0.007
V_10_ (%)	46.51±11.7	61.1±14.5	0.000
V_15_ (%)	33.6±5.8	41.9±8.4	0.000
V_20_ (%)	25. 9±2.5	29.6±1.6	0.000
V_25_ (%)	21.4±2.3	23.5±2.1	0.007
V_30_ (%)	17.9±2.3	18.9±2.6	0.227
V_35_ (%)	14.6±2. 6	14.5±3.0	0.954
V_40_ (%)	11.0±2.9	10.2±3.4	0.355
V_45_ (%)	7.0±2.9	5.7±3.4	0.181
PTV(V/cm^3^)	882.6±335.1	931.8±282.6	0.655
Ipsilateral lung volume(V/cm^3^)	1230.4±294.3	1339.9±385.8	0.279
Bilateral lung volume(V/cm^3^)	2444.3±592.0	2798.7±702.3	0.079
The maximum dose of the lungs (D/Gy)	55.7±3.0	55.7±2.5	0.949
The average dose of bilateral lung (D/Gy)	8. 6±1.5	9.4±1.1	0.101
The maximum dose of contralateral lung (D/Gy)	16.8±14.9	18.5±13.6	0.726
The average dose of contralateral lung (D/Gy)	1.8±2.2	1.8±0.9	0.922
The maximum dose of ipsilateral lung (D/Gy)	55.6±2.9	55.5±2.3	0.928
The average dose of ipsilateral lung (D/Gy)	14.8±1.6	17.0±1.1	0.000

### Multivariate logistic regression analysis and ROC curve

**Figure 1:**
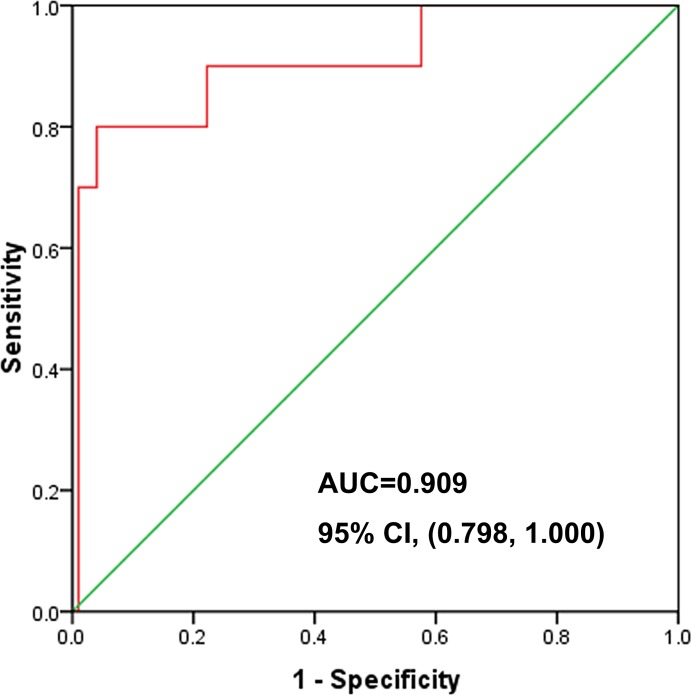
ROC curve analysis of V_20_ AUC = 0.909, 95% CI (0.798, 1.000).

Logistic regression analysis showed that V_20_ determines the independent risk factors of RILI (OR = 2.618, OR value of 95% CI 1.447 to 4.737), Table 3. ROC curve analysis suggested that ipsilateral lung V_20_ could predict RILI with an area under the ROC curve (AUC) of 0.909. The AUC of 95% CI was 0.798 to 1.000, and V_20_ of 29.03% was the best cut-off point; the sensitivity of the prediction of RILI was 80%, and the specificity was 96% (Figure 1).

**Table 3:** Odds ratio and 95% CI for RILI from multivariate analysis of 109 breast cancer patients

Factors	*P value*	OR	95%CI
V5 (%)	0.471	1.051	0.919-1.202
V10(%)	0.531	1.060	0.883-1.273
V15(%)	0.965	1.006	0.784-1.289
V20(%)	0.001	2.618	1.447-4.737
V25(%)	0.995	1.004	0.318-3.172
The average dose of ipsilateral lung (Gy)	0.462	0.628	0.181-2.173
The number of cycles of chemotherapy	0.076	8.488	0.798-90.317

### Model parameters for Lyman NTCP

**Figure 2:**
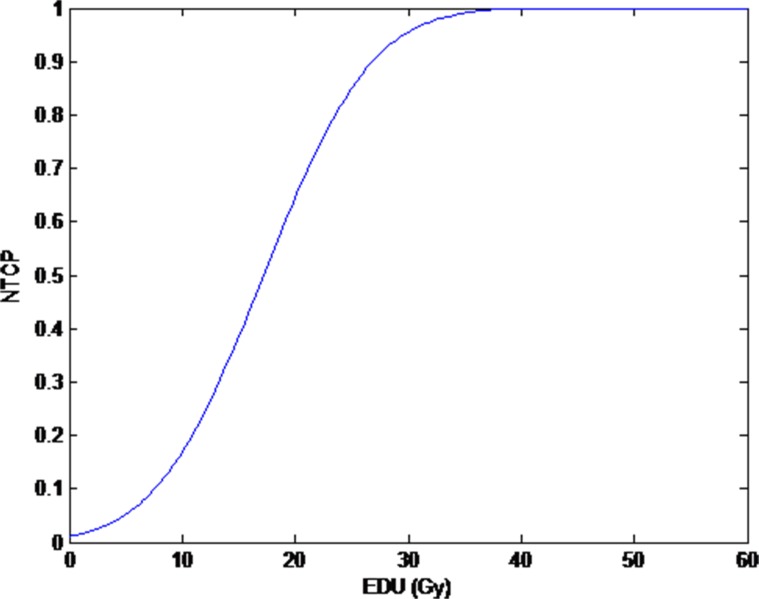
The relationship between NTCP and Mean dose to ipsilateral lung in 109 breast cancer cases (*n* = 0.912, m = 0.437, TD50(1) = 17.211 Gy) EUD: equivalent uniform dose.

The model parameters that were calculated in the 109 cases of breast cancer for Lyman NTCP were n = 0.912, m = 0.437, and TD50 (1) = 17.211 Gy. The relationship between the mean dose and NTCP in the 109 breast cancer patients in the ipsilateral lung is shown in Figure 2.

### ROC curve of NTCP

**Figure 3:**
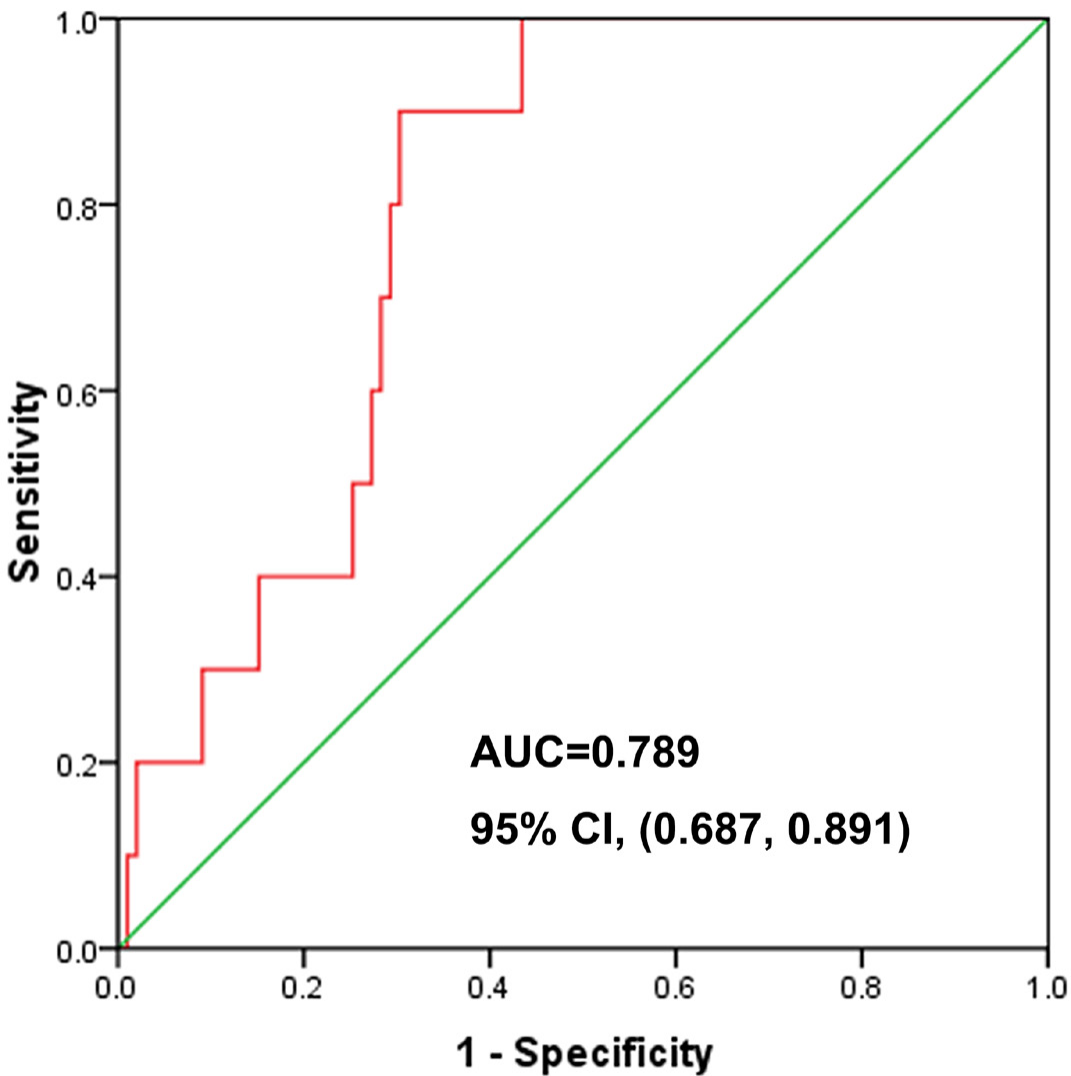
One hundred and nine cases of postoperative breast cancer patients after IMRT; ROC curve of NTCP, each of which had a projected NTCP value using the NTCP model (*n* = 0.912, m = 0.437, TD_50_ (1) = 17.211 Gy), AUC = 0.789, 95% CI (0.687, 0.891)

As shown in Figure 3, the ROC curve from 109 breast cancer patients had an IMRT NTCP value with AUC of 0.789, 95% CI (0.687, 0.891), with NTCP = 9.62% as the best cut-off value; the sensitivity of the prediction model of RILI was 0.900 (9/10), the specificity was 0.697 (69/99), and the accuracy was 0.716 (78/109). The positive predictive value was 0.230 (9/39) and the negative predictive value was 0.986 (69/70). In 109 cases of breast cancer, NTCP less than 9.62% patients with breast cancer had an incidence of RILI of 1.43% (1/70), which is far lower than in the NTCP more than 9.62% breast cancer patients with RILI, who had an incidence of 23.08% (9/39); there was significant difference between two arms (*p* = 0.001), Figure 3.

## DISCUSSION

RILI includes early acute radiation pneumonia and late chronic radiation pulmonary fibrosis [9]. Clinical data suggested that the whole lung irradiation dose could not exceed 20 Gy; the ipsilateral lung V_20_ less than 30% is safe [14]. Some studies suggested that the occurrence of RILI is associated with the use of chemotherapy and tamoxifen [11, 15, 16]. Some prospective studies found that lung injury is associated with age and individual radiation susceptibility [12, 17]. Other studies have reported that smoking may reduce the incidence of RILI [18]. Our study focuses on the dosimetric parameters and Lyman NTCP model related to RILI to predict the incidence of RILI, optimize the radiotherapy plan and improve the patients’ quality of life. The study group patients did not have smoking history and took no endocrine drugs during the radiotherapy period. The study results show that age has no effect on the occurrence of RILI (*P* > 0.05). Considering the effect of a small number of cases or short follow-up time, the clinical staging and operation effect on RILI manifested no statistical significance.

The lung V_20_, mean dose of normal lung (MLD) and other dosimetric parameters are predictive of RILI. When MLD≤17 Gy, V_20_≤31.0%, and V_30_≤24.0%, the probability of severe RILI will be 0-10.7% [19, 20]. Claude et al. [21] reported that for MLD, V_20_ for level 1 and level 1 above a meaningful forecast, RILI, MLD and V_20_ are associated with grade 2 or greater RILI. Graham et al. [22] found that single factor analysis suggests that the discretion of the incidence of RILI and severity are closely associated with irradiated lung volume and dose. Multivariable analysis found that V_20_ is the most important factor that affects RILI; when V_20_ < 22%, no RILI occurs, when V_20_ is between 22% and 31%, 32% and 40% and > 40%, the 24 month RILI incidence is 7%, 13% and 7%, respectively. At the same time, when V_20_ is between 22% and 31%, 8% of patients have 2nd level RILI, while 3rd level radioactive pneumonia occurs when V_20_ ≥ 32%. Asakura et al. [23] found that V_20_ is an independent risk factor in patients with oesophageal synchronous chemotherapy; when V_20_ < 24%, the incidence of RILI is 13%, when 25% < V_20_ < 36%, the rate is 33% and when V_20_ ≥ 37%, the incidence is 78%. Additionally, some scholars demonstrated that in normal lungs, MLD and the occurrence of RILI also showed a positive correlation; here, with the increase of MLD, the incidence of RILI also gradually increased. Roeder et al. [24] found that when the MLD < 10 Gy, the incidence of RILI was 7%; when 10 Gy < MLD < 20 Gy, the incidence of RILI was 19%; and when MLD > 20 Gy, the incidence of RILI increased significantly. Therefore, V20 and Dmean should be chosen to measure the parameters of RILI. Univariate factor analysis shows that the RILI in chemotherapy cycle number, the average dose of lung and V_5_-V_25_ had obvious differences (*p* < 0.05); the logistic regression analysis showed that V_20_ had the closest relationship with RILI. When V_20_ > 29.03%, the incidence of RILI was 66.67%, and V_20_ acuities were 29.03% when the incidence of RILI was 2.06%; the difference between the two was statistically significant (*p* = 0.000). The results suggest that V_20_ can serve as a predictor of RILI occurrence and evaluate the merits of the radiotherapy plans. Therefore, according to V_20_≤29.03%, the parameter values for predicting the occurrence of RILI can effectively reduce the incidence of RILI and improve the quality of life in breast cancer patients.

The lung injury induced by chemotherapy drugs and RILI can produce superimposed effects [25]. Theuws et al. found that patients with breast cancer receiving chemotherapy plus radiotherapy in the application of CMF showed more of a decline in lung function than with simple radiotherapy, with a significant result [26]. Dang et al. analysed 93 RILI patients with non-small cell lung cancer; in combined radiation and chemotherapy patients, the incidence of RILI was 61.1%, which was significantly higher than the 42.1% reported in radiation therapy alone [27]. Our data analysis showed that the incidence of lung injury in the chemotherapy group undergoing > 6 cycles is higher than in the group receiving 6 or fewer chemotherapy cycles (*p* < 0.05). This study does not provide a detailed analysis of the impact of different chemotherapy regimens on RILI because the selected cases were included for various reasons (including an inability to tolerate side effects during the chemotherapy period, and personal economic reasons meaning that the patient could not afford to complete chemotherapy cycles). However, chemotherapy failed to enter the multivariable analysis. The logistic regression model is the main reason that the research analysis may have not considered the interactions between all factors, ignoring the influence between them.

The Lyman NTCP model is derived from the DVH graph for mathematics-related data to calculate the probability of complications after radiotherapy in patients. In 1991, Burman found the Emani data value fitting model parameters n, m, and TD_50_ of the lung values to be 0.87,0.18, and 24.5 Gy, which have a large volume effect on RILI [28]; this also means that NTCP relies more on the whole volume according to the average dose. For the study of RILI, Lyman NTCP model parameters showed some differences in the patient population and disease; as a result, the calculated parameters are not the same, but generally reflect the effect of lung volume, and n value is 0.87 to 1.0. Shi et al. analysed 94 patients with locally advanced non-small cell lung cancer [29]; for the resulting NTCP multiple factors, regression analysis found that NTCP was 4.2%, which was used as a cut-off value point. When NTCP acuities were 4.2% and > 4.2%, the incidence of RILI was 1.4% and 43.5%, respectively. Using maximum likelihood method fitting, this study collected the new parameter of the model as *n* = 0.912, m = 0.437, TD_50_(1) = 17.211 Gy, and NTCP = 9.62% as the diagnostic values; the model to predict the sensitivity of RILI was 0.9(9/10), with a specific degree of 0.697(69/99), and an accuracy of 0.716 (78/109). In 109 breast cancer patients, NTCP < 9.62% of patients showed an incidence of RILI of 1.43% (1/70), which was far lower than the NTCP ≥ 9.62% of breast cancer patients, who had a RILI incidence rate of 23.08% (9/39), suggesting that the model had a good prediction effect on RILI. Tsougos analysed the NTCP model and found its predictive value in non-small cell lung cancer patients with RILI [30]; if the calculation of the ipsilateral lung NTCP, and the clinical results of NTCP model on the value of the (RTOG grade 2 RILI) are in good agreement, and the left and right side of the lung as a whole to calculate the value of NTCP is calculated, values of the model are not very good with regard to lung injury by clinical observation, and the clinical incidences underestimated. All of the cases were collected along with dosimetry data for ipsilateral breast tumour of the lung; it did not compare the contralateral and bilateral pulmonary NTCP values.

In conclusion, NTCP could be used for the evaluation of the radiotherapy target in the optimization of IMRT. The Lyman NTCP model parameters of the new value (m = 0.437, *n* = 0.912, TD_50_(1) = 17.211 Gy) can be used as an effective index to evaluate the risk of occurrence of RILI. In addition, V_20_ was an independent predictive factor for RILI in patients with breast cancer treated by IMRT. V_20_ = 29.03% could be a useful dosimetric parameter for evaluating the risk of RILI.

## MATERIALS AND METHODS

### Patients and inclusion criteria

The study prospectively included all of the female breast cancer patients who were treated after surgery in the Cancer Hospital of Guangxi Medical University between January 2012 and December 2013. Inclusion criteria: (1) female patients who underwent breast cancer surgery in the hospital with breast cancer confirmed by postoperative pathology diagnosis; (2) age from 25 to 75 years old; (3) Karnofsky score (Karnofsky performance status, KPS) ≥ 70 points; (4) no smoking history; (5) patients who underwent IMRT for the first time; (6) no concurrent chemotherapy and endocrine therapy during radiotherapy; and (7) patients completed radiotherapy and were followed-up for more than 6 months. Exclusion criteria: (1) patients with tumour recurrence or distant metastasis; (2) patients with concurrent heart or lung disease; (3) patients who could not tolerate radiotherapy or failed to complete radiation therapy for any reason. This study was approved by the Institutional Review Board of Cancer Hospital of Guangxi Medical University, which required informed consent.

### Treatment methods

IMRT was performed using a vacuum pad in a fixed position. The CT scan imaging system was networked with the radiotherapy planning system, delineating clinical target volume (CTV) and organ at risk (OAR), including spinal cord, heart, lung and contralateral breast. The planning target volume (PTV) is an extension of CTV. The dose of radiotherapy after breast-conserving surgery was as follows: whole breast irradiation with 6 MV X-rays, with a total dose of 50 Gy/5 W (2 Gy/f·d^−1^, 5f/W). The supraclavicular region was used, and exposure of the whole breast was prevented with axillary equipment. The breast tumour bed synchronous dosage was 10-16 Gy/1-1.5 W (2 Gy/f·d^−1^, 5f/W) in patients with invasive breast cancer and negative margins; the breast tumour bed boost was 16-20 Gy/1.5-2 W (2 Gy/f·d^−1^, 5f/W) in patients with invasive breast cancer and positive margins. After modified radical mastectomy for breast cancer, the radiotherapy dose was as follows: using 6 MV X-ray irradiation (2 Gy/f, 50 Gy/5 W·d^−1^, 5f/W). All patients who underwent modified radical mastectomy had treatment of the chest wall surface using a 0.5 cm thick film pad when irradiated equivalent to 20 Gy, and then the equivalent film was removed when irradiated to 50 Gy. The treatment plan required that 95% of the target dose covered the target volume of 100%, and the maximum dose was less than 110% of the prescribed dose. The PTV dose and organ damage limits were: ipsilateral lung, V_20_ < 30%, D_mean_ < 20 Gy; bilateral lung, V_20_ < 20%; heart (only left-sided breast cancer patients), V_30_ < 10%, V_40_ < 5%; and contralateral breast, D_mean_ < 1 Gy, D_max_ < 5 Gy.

### Diagnosis and evaluation of RILI

According to the American Radiation Therapy Oncology Group (radiation therapy, oncology group, RTOG), acute and chronic RILI can be divided into 5 levels based on standard RILI evaluation [31]. After radiotherapy, following up for 1 to 6 months and for more than 6 months was used to diagnose acute radiation pneumonia and chronic RILI.

### Clinical and physical parameters

RILI-related clinical factors such as age, clinical operation, tumour stage, number of chemotherapy cycles and physical parameters, including the planning target volume (PTV), ipsilateral lung and bilateral lungs volume, bilateral lungs, ipsilateral lung and contralateral lung maximum dose, mean dose, and lung V_5_, V_10_, V_15_, V_20_, V_25_, V_30_, V_35_, V_40_, and V_45_ (i.e., the ipsilateral lung received 5, 10, 15, 20, 25, 30, 35, 40, and 45 Gy dose of the lung volume to the total lung volume percentage) were prospectively collected.

### The model of Lyman NTCP

The Dose Volume Histogram (DVH) data for each patient were transferred to MATLAB (version R2009b, MathWorks, USA) software. The data were fitted using the Lyman-Kutcher-Burman (LKB) NTCP model [10, 13, 32]. The Lyman NTCP model formula is as follows [32]:

$$\text{NTCP}=\theta \left(t\right)=\frac{1}{\sqrt{2\pi }}{\int_{-\infty }^{t}e}^{-\frac{x^{2}}{2}}dx,\;t=\left(\frac{D-TD_{50}\left(v\right)}{m\cdot TD_{50}\left(v\right)}\right),\;TD_{50}\left(v\right)=TD_{50}\left(1\right)\cdot v^{-n}$$

The Lyman NTCP model only considers uniform illumination; the radiation dose of normal lung radiotherapy in breast cancer is not uniform, with Kutcher-Burman equivalent volume calculation method used to normalize non-uniform dose-distribution (DVH plots) converted into equivalent uniform distribution [10]. An equivalent volume is defined as the volume of irradiated lung tissue that receives a considerable probability of lung injury due to uniform illumination and the real situation of uneven irradiation. The most widely used NTCP model for radiation pneumonitis (RP) is the LKB model. This model has three parameters, a position parameter, TD_50_, a steepness parameter, *m*, and the volume exponent, *n* (where *n* = 1 the model reverts to mean lung dose; MLD). While TD_50_ is strongly dependent on the grade of RP being considered, n is often regarded as a tissue characteristic. The Newton-Raphson method was used to calculate m, n, TD_50_ and maximum likelihood parameter estimation by MATLAB [10, 13, 32].

### Statistical analysis

SPSS22.0 (IBM SPSS, NY, USA) statistical software was used for analysis. Univariate analysis using the t test and χ^2^ test and multivariate analysis using the logistic regression model. Receiver operating characteristic (ROC) curve analysis of ipsilateral lung volume percentage was in relation to the RILI. In each case, the NTCP value with the Lyman-Kutcher-Burman model was calculated; the relationship between the parameters and the occurrence of RILI was analysed using the t test, and the difference between the two groups in the model parameters was compared using the t test. Receiver operating characteristic (ROC) curve analysis of the new Lyman model was performed to calculate the relationship between the NTCP value and RILI. *P* < 0.05 was considered statistically significant.
